# A Randomized Study to Compare the Effects of Once-Weekly Dulaglutide Injection and Once-Daily Glimepiride on Glucose Fluctuation of Type 2 Diabetes Mellitus Patients: A 26-Week Follow-Up

**DOI:** 10.1155/2019/6423987

**Published:** 2019-04-30

**Authors:** Huiqin Li, Xiaohua Xu, Jie Wang, Xiaocen Kong, Maoyuan Chen, Ting Jing, Zhiying Zhang, Guoping Yin, Xiaomei Liu, Yun Hu, Lei Ye, Xiaofei Su, Jianhua Ma

**Affiliations:** ^1^Department of Endocrinology, Nanjing First Hospital, Nanjing Medical University, Nanjing 210012, China; ^2^National Heart Research Institute Singapore, National Heart Centre Singapore, Singapore

## Abstract

**Objective:**

To evaluate the effects of once-weekly dulaglutide injection and once-daily glimepiride on glucose fluctuation in patients with type 2 diabetes mellitus (T2DM) using the Continuous Glucose Monitoring System (CGMS).

**Methods:**

A total of 23 patients with T2DM were randomly assigned into two groups for 26 weeks: the dulaglutide group (*n* = 13) and the glimepiride group (*n* = 10). 72-hour CGMS was applied to all patients: before and after the treatment. General clinical data were collected and measured, such as fasting blood glucose (FBG), glycosylated hemoglobin (HbA1c), tumor necrosis factor-*α* (TNF-*α*), 8-*iso*-prostaglandin F2*α* (8-*iso*-PGF2*α*), and interleukin-6 (IL-6).

**Results:**

HbA1c of the dulaglutide group was reduced from 8.38 ± 0.93% to 6.68 ± 0.73% after the treatment (*P* < 0.05); similarly, it was reduced from 7.91 ± 0.98% to 6.67 ± 0.74% (*P* < 0.05) in the glimepiride group. The levels of serum 8-*iso*-PGF2*α*, TNF-*α*, and IL-6 all decreased significantly in both groups after treatment, and there was no significant difference found between the two groups (*P* > 0.05). The Mean Blood Glucose (MBG) of the two groups declined significantly after therapy (*P* < 0.05). However, the Standard Deviation of Blood Glucose (SDBG) decreased significantly only in the dulaglutide group (from 2.57 ± 0.74 mmol/L to 1.98 ± 0.74 mmol/L, *P* < 0.05). There were no significant changes of Mean Amplitude of Glycemic Excursion (MAGE) and Absolute Means of Daily Difference (MODD) after treatment in both groups. Furthermore, no statistically significant difference was found between the two groups in MBG, SDBG, MAGE, and MODD (*P* > 0.05). The percentage time (PT) (>10 mmol/L and 3.9-10 mmol/L) of the two groups was significantly changed after the treatment (*P* < 0.05). However, this was not seen in the PT < 3.9 mmol/L after the treatment (*P* > 0.05).

**Conclusion:**

Once-weekly dulaglutide injection has the same effectiveness as daily glimepiride on lowering blood glucose and decreasing oxidation stress and inflammation and is more effective in controlling glucose fluctuation as compared with glimepiride. This trial is registered with ClinicalTrials.gov NCT01644500.

## 1. Introduction

The prevalence rate of type 2 diabetes mellitus (T2DM) is rising in the world. It has been one of the most common chronic metabolic disorders and has been a big burden for patients, especially for those in developing countries [1]. The prevalence rate of diabetes in China so far had increased to 11.6% [2]. China has been the country that has the largest number of diabetic patients in the world [1].

Dulaglutide (once-weekly injection) is a long-acting glucagon-like peptide-1 (GLP-1) analog. It is more effective in improving patient adherence [3] than short-acting GLP-1 preparations (1-3 injections per day). Many studies suggest that dulaglutide is effective in lowering the patients' glycosylated hemoglobin (HbA1c) [4–6]. However, few studies have investigated the effects of dulaglutide on glucose fluctuation. It is known that glucose fluctuation has a more severe effect on oxidative stress and prognosis of cardiovascular diseases than persistent chronological high blood glucose [7–11]. Thus, the present study chose the T2DM patients who had never received antidiabetic drugs or who were receiving oral antihyperglycemic medication (OAM) monotherapy as subjects and was aimed at determining the effect of dulaglutide on glucose fluctuations for 26 weeks in comparison with glimepiride. The Continuous Glucose Monitoring System (CGMS) was applied in all patients to record and evaluate glucose fluctuations before and after the treatment.

## 2. Materials and Methods

### 2.1. Subjects

The study was approved by the ethics committee of Nanjing Hospital. It was registered with ClinicalTrials.gov: NCT01644500. All procedures followed were in accordance with the Helsinki Declaration of 1964, as revised in 2013. Informed consent was obtained from all patients for being included in the study. The patient inclusion criteria were as follows: (1) T2DM patients who had never received OAM (7.0%≤HbA1c ≤ 10.5%) or who were receiving OAM monotherapy (6.5%≤HbA1c ≤ 10.0%, the daily dose of oral antidiabetic drug intake must be less than 50% of the recommended dose), (2) the body mass index (BMI) levels of all the patients being between 19.0 and 35.0 kg/m^2^, and (3) patients that were adult males or nonbreastfeeding females who were older than 18 years.

The exclusion criteria were as follows: (1) patients had type 1 diabetes; (2) patients had been treated with either a GLP-1 receptor agonist or a GLP-1 analogue or were taking dipeptidyl peptidase-4 (DPP-IV) inhibitor, thiazolidinedione, or insulin; (3) patients had severe diabetic gastroparesis or had been taking drugs that directly affect gastrointestinal motility for the long term; and (4) patients had either of the following diseases: acute or chronic hepatitis, acute or chronic pancreatitis, or abnormal renal function, had a serum calcitonin concentration higher than 20 pg/mL, or had a history of malignancy.

Twenty-three patients with T2DM were included in the study during the period of Dec. 2012 and Aug. 2013 at the Nanjing First Hospital, Nanjing, China. They were randomly assigned into two groups: the once-weekly dulaglutide injection group and the once-daily glimepiride oral group. Among these subjects, 13 subjects (6 females and 7 males) were in the dulaglutide group and 10 subjects (4 females and 6 males) in the glimepiride group. There were 8 cases (0.75 mg dulaglutide) and 5 cases (1.5 mg dulaglutide). Patient adherence was 100% in both groups. No SAE occurred in this study. Abdominal distension occurred in 3 cases in the dulaglutide group and loss of appetite in 1 case in the glimepiride group.

### 2.2. Methods

Patients' clinical data were collected. Fasting blood glucose (FBG), HbA1c, and biochemical indexes were measured and recorded by a specially assigned physician. The starting dosage of glimepiride was 1 mg, and the dosage could be adjusted on week 4 or 8, based on the patient's glucose level. For those who were taking antidiabetic drugs, they would spend two weeks after passing the screening procedure as the washing-out period before receiving the treatments.

For CGMS measurement, a 72-hour CGMS (Medtronic MiniMed, USA) was applied to all subjects before and at week 26 of the treatment. The subjects were required to record their time of eating and monitor and record their finger peripheral blood glucose at least 4 times/day and hypoglycemic episodes (blood glucose < 3.9 mmol/L). They were instructed to take food when their blood glucose was lower than 3.9 mmol/L.

Indexes recorded and measured by CGMS included (1) Mean Blood Glucose (MBG); (2) Standard Deviation of Blood Glucose (SDBG); (3) Mean Amplitude of Glycemic Excursion (MAGE): 24-hour amplitude variations of the subjects' glycemic excursion were measured and statistically analyzed, with the mean amplitude value of all glycemic variations calculated; (4) Absolute Means of Daily Difference (MODD): corresponding values of the subjects' blood glucose in the two successive 24 hours, which were measured and recorded at the same time stage of the day, were compared, with differences between the two being calculated and a mean value of the difference obtained; and (5) percentage time (%, PT): using 2.8 mmol/L, 3.9 mmol/L, 10.0 mmol/L, and 13.9 mmol/L of blood glucose as points of tangency, their percentages of 24 hours were calculated.

Tumor necrosis factor-*α* (TNF-*α*), 8-*iso*-prostaglandin F2*α* (8-*iso*-PGF2*α*), and interleukin-6 (IL-6) were measured in serum as previously described [12].

### 2.3. Statistical Methods

The blood glucose-changing profile was collected and analyzed using CGMS software 3.0. Statistical analysis was conducted using SPSS 20.0. The continuous variables were expressed as mean ± standard deviation (SD). Enumeration data was analyzed by chi-square test. Comparisons between the two groups were done with *t*-test, and nonparametric analysis would be used when heterogeneity of variance was found. Differences of *P* < 0.05 were considered statistically significant.

## 3. Results

### 3.1. Patient Baseline Demographics

Thirteen subjects (6 females and 7 males) were included in the dulaglutide group and 10 subjects (4 females and 6 males) were included in the glimepiride group. The mean age of the dulaglutide and glimepiride groups was 54.75 ± 5.33 years and 53.30 ± 6.60 years, respectively. Those who had received antidiabetic drugs were 7 in the dulaglutide group and 6 in the glimepiride group (*P* > 0.05) (Table 1).

### 3.2. Clinical Data when Being Treated for 26 Weeks

The HbA1c of the dulaglutide group decreased from 8.38 ± 0.93% to 6.68 ± 0.73% after the treatment (*P* < 0.001). It decreased from 7.91 ± 0.98% to 6.67 ± 0.74% after the treatment (*P* = 0.008) in the glimepiride group.

### 3.3. Oxidative Stress and Inflammatory Profile

To determine the effect of dulaglutide and glimepiride on oxidative stress and inflammation, we measured the serum levels of 8-*iso*-PGF2*α*, TNF-*α*, and IL-6. Compared to baseline, the levels of serum 8-*iso*-PGF2*α*, TNF-*α*, and IL-6 were significantly decreased in both groups after treatment, and there was no significant difference on the reduction of oxidative stress and inflammation between the two drugs (Tables 2 and 3).

### 3.4. Comparison of CGMS Glucose Fluctuation Profiles

**Figure 1:**
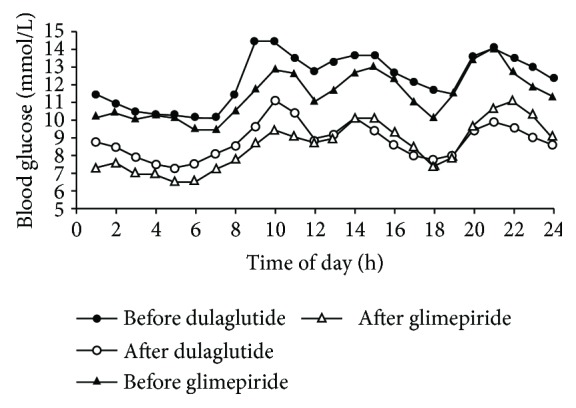
24 h CGMS glucose fluctuation profile of patients having dulaglutide or glimepiride.

**Figure 2:**
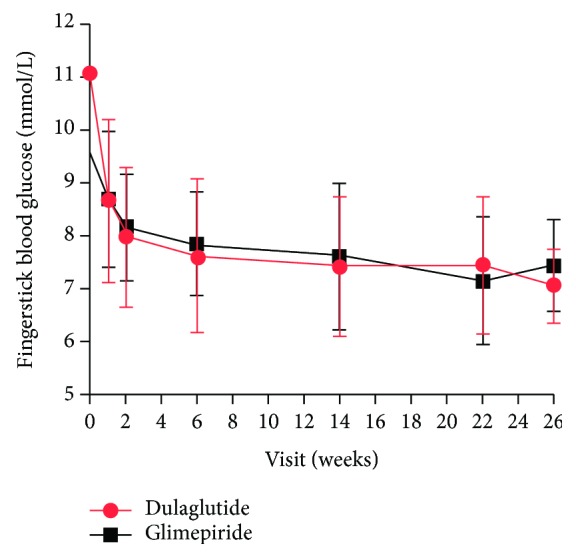
Fingerstick BG of two groups from 0 week to 26 weeks.

The patients' 24 h CGMS glucose profile is shown in Figure 1. Fingerstick BG of two groups from 0 week to 26 weeks is shown in Figure 2. The MBG of the two groups decreased significantly after the treatment (*P* < 0.05). However, there were no statistically significant differences between the two groups (*P* > 0.05).

The SDBG declined significantly only in the dulaglutide group (from 2.57 ± 0.74 mmol/L to 1.98 ± 0.74 mmol/L after the treatment, *P* < 0.05). There was no statistically significant difference between the two groups (*P* > 0.05). There were no statistically significant changes of MAGE and MODD found at baseline or 26 weeks after treatment (*P* > 0.05).

**Figure 3:**
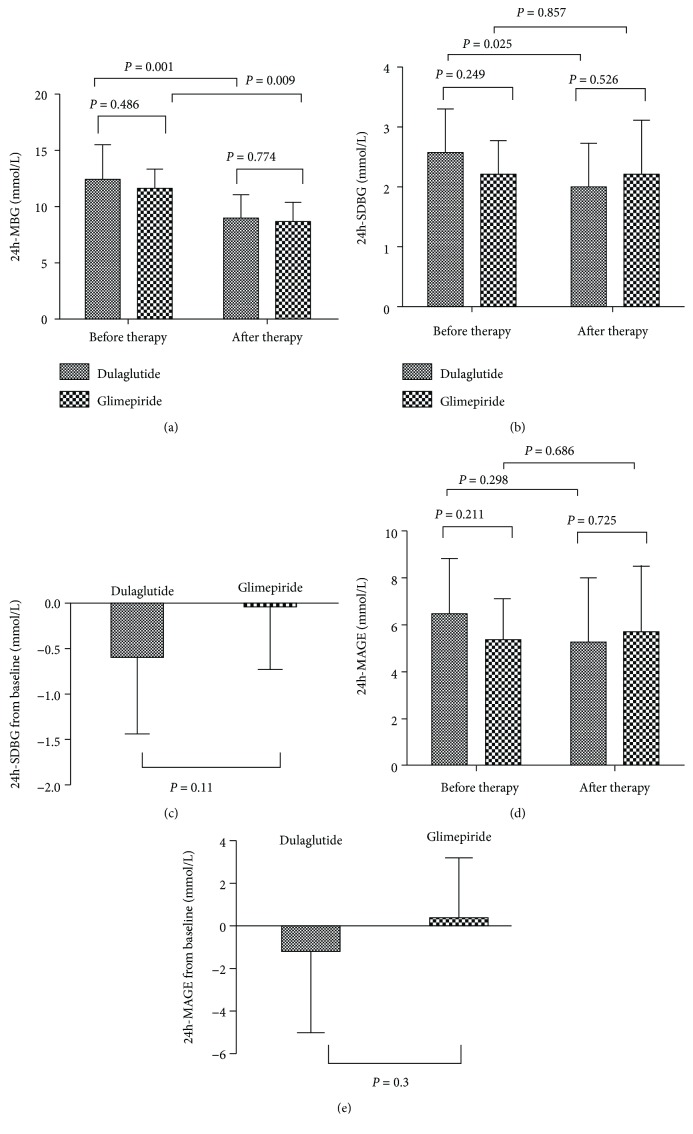
Comparisons of CGMS glucose parameters: (a) 24h-MBG, (b) 24h-SDBG, (c) 24h-SDBG, (d) 24h-MAGE, and (e) 24h-MAGE at baseline and after treatment in the dulaglutide and glimepiride groups.

There was statistical significance in the PT (>10 mmol/L reduction, 3.9-10 mmol/L increment) of the two groups before and after the treatment (*P* < 0.05). There was no statistical significance, however, in the PT (<3.9 mmol/L) before and after the treatment (*P* > 0.05). There was no statistical intergroup significance in the PT of the two groups (>10 mmol/L or <3.9 mmol/L) (*P* > 0.05) (Tables 2 and 3 and Figure 3).

## 4. Discussion

It is found that both dulaglutide and glimepiride can effectively reduce HbA1c, MBG, and PT (>10 mmol/L) as detected by CGMS and decrease oxidation stress to similar levels. Once-weekly dulaglutide injection can more effectively improve blood glucose fluctuation compared with glimepiride.

Dulaglutide is a kind of long-acting GLP-1 analog and has been proved by studies to have the effect of improving patients' glycemic control [4–6, 13–15]. Actually, dulaglutide has better effects on lowering blood glucose and HbA1c than saxagliptin, exenatide, or metformin [4–6, 13]. It also can control postprandial glucose by improving the function of the pancreas islet [15]. It is not only tolerated by older patients (>65 years) but can also achieve the same therapeutic effects of lowering their blood glucose as with younger patients. These indicate that dulaglutide is a safe and more effective antidiabetic drug even for aged diabetic patients [14].

Several studies indicated that in addition to lowering glucose, dulaglutide had an effect on the cardiovascular system [16–18]. Ferdinand et al.'s study of 755 patients with T2DM found that dulaglutide could lower systolic blood pressure for 2.8 mmHg compared with placebos [16]. Tuttle et al.'s study of 6005 patients with T2DM showed that dulaglutide could slightly lower urine protein, but did not lower the glomerular filtration rate. Furthermore, Ferdinand et al.'s study demonstrated that dulaglutide did not increase major adverse cardiac events [17, 18].

Although a number of studies had suggested that both dulaglutide and glimepiride could effectively reduce blood glucose [4–6, 13–15], there was no head-to-head study on the effect of dulaglutide and glimepiride using CGMS. The current study showed that MBG and HbA1c of both the dulaglutide and glimepiride groups decreased significantly after the treatment (*P* < 0.05). The amplitudes of MBG reduction of the two groups were similar. The PT results also showed that the changing profile (reduction of percentages of PT > 10 mmol/L and increment of percentage of PT between 3.9 and 10 mmol/L) of both groups was also similar, indicating that both dulaglutide and glimepiride could lower blood glucose effectively.

Recently, glucose fluctuations have been found to have greater effect on oxidative stress and prognosis of cardiovascular disease than persistent chronic hyperglycemia [7–11]. Intermittent hyperglycemia, therefore, could be an independent factor for the occurrence of chronic diabetic complications with the exception of the high level of mean glucose [19]. Few studies evaluated the effects of dulaglutide on glucose fluctuations, and head-to-head comparison on the effects of dulaglutide and glimepiride on glucose fluctuations is limited. AWARD-4 by Jendle et al. applied CGMS to 144 patients with T2DM by dulaglutide or glargine combined with insulin lispro and found that the SD of the dulaglutide group decreased significantly than that of the glargine group after being treated for 26 weeks and the intergroup difference had statistical significance. Furthermore, the MODD of the dulaglutide group was significantly lower than that of the glargine group. These studies indicate that the glucose fluctuation after dulaglutide treatment was lower than that after the glargine treatment [20].

In the present study, the SDs of the dulaglutide group were significantly reduced, while they were almost unchanged in the glimepiride group after the 26-week treatment. The MODDs of both groups had a trend to reduce after treatment. These indicated that dulaglutide may have better control on blood glucose fluctuations.

Our results showed that dulaglutide and glimepiride therapies also decreased oxidation stress and inflammation markers in T2DM patients including 8-*iso*-PGF2*α*, TNF-*α*, and IL-6. This confirms that GLP-1 has anti-inflammatory activity [21], which may be related to the reduction in glucotoxicity.

Risk of hypoglycemia episodes was a barrier that prevented antidiabetic drugs from being used. Using 2.8 mmol/L and 3.9 mmol/L of blood glucose, as points of tangency, we found that there were no statistically significant differences in PTs lower than 3.9 mmol/L or lower than 2.8 mmol/L before or after treatment. This indicates that the once-weekly dulaglutide injection will not increase incidence of hypoglycemia as compared with daily glimepiride.

In conclusion, once-weekly dulaglutide injection has the same effectiveness as daily glimepiride on lowering blood glucose and decreasing oxidation stress and inflammation and is more effective on controlling glucose fluctuation as compared with glimepiride. Because it only needed 1 injection per week, it could improve the adherence of patients and had a good clinical application prospect. Dulaglutide therefore would be a new alternative for treating diabetes due to its effectiveness, safety, and convenience.

## Figures and Tables

**Table 1:** Characteristics of patients.

	Dulaglutide	Glimepiride	*P*
Cases (*n*)	13	10	
Male/female (*n*)	7/6	6/4	0.768
Age (years)	54.75 ± 5.33	53.3 ± 6.60	0.575
Diabetes history (years)	2.0 (0.5, 9.0)	2.0 (1.0, 3.5)	0.851
Body weight (g)	66.85 ± 9.04	66.60 ± 8.56	0.948
BMI (kg/m^2^)	23.98 ± 2.5	24.78 ± 2.64	0.466
Systolic blood pressure (mmHg)	128.15 ± 14.80	127.2 ± 10.65	0.865
FBG (mmol/L)	11.12 ± 2.63	9.59 ± 1.32	0.09
HbA1c (%)	8.38 ± 0.93	7.91 ± 0.98	0.25
Cholesterol (mmol/L)	5.48 ± 1.07	4.31 ± 0.77	0.008^∗^
HDL cholesterol (mmol/L)	1.33 ± 0.29	1.11 ± 0.48	0.181
LDL cholesterol (mmol/L)	3.09 ± 0.90	2.49 ± 0.68	0.089
Triglycerides (mmol/L)	2.27 ± 1.03	1.39 ± 0.73	0.031^∗^
Hemoglobin (g/L)	149.54 ± 11.44	144 ± 18.04	0.379
Urea nitrogen (mmol/L)	6.24 ± 1.55	5.21 ± 1.77	0.156
Creatinine (*μ*mol/L)	74.62 ± 15.68	71.2 ± 18.96	0.641
AST (U/L)	22.38 ± 9.32	19.1 ± 4.82	0.323
Uric acid (*μ*mol/L)	297.46 ± 48.87	301.6 ± 50.01	0.844
GFR (mL/min)	82.08 ± 19.46	98.8 ± 26.12	0.093

BMI: body mass index; FBG: fasting blood glucose; HbA1c: glycosylated hemoglobin; HDL: high-density lipoprotein; LDL: low-density lipoprotein; AST: aspartate transaminase; GFR: glomerular filtration rate. Data shown as mean ± SD or median (first quartile, third quartile). ^∗^Dulaglutide vs. glimepiride: *P* < 0.05.

**Table 2:** Comparison of blood fluctuation, oxidative stress, and inflammatory profile within groups.

	Dulaglutide			Glimepiride		
	Baseline	26 weeks after treatment	*P*	Baseline	26 weeks after treatment	*P*
FBG (mmol/L)	11.12 ± 2.63	7.77 ± 1.33	<0.001	9.59 ± 1.32	7.45 ± 0.95	0.005
HbA1c (%)	8.38 ± 0.93	6.68 ± 0.73	<0.001	7.91 ± 0.98	6.67 ± 0.74	0.008
Body weight (kg)	66.85 ± 9.04	66.62 ± 8.84	0.704	66.60 ± 8.56	67.80 ± 8.67	0.22
MBG (mmol/L)	12.35 ± 3.23	8.84 ± 2.21	0.001	11.53 ± 1.88	8.59 ± 1.75	0.009
SDBG (mmol/L)	2.57 ± 0.74	1.98 ± 0.74	0.026	2.24 ± 0.54	2.2 ± 0.92	0.857
MAGE (mmol/L)	6.49 ± 2.33	5.32 ± 2.6	0.298	5.35 ± 1.74	5.73 ± 2.82	0.686
MODD (mmol/L)	2 ± 0.59	1.71 ± 0.80	0.336	2.53 ± 0.89	1.85 ± 0.75	0.075
PT < 2.8 (mmol/L)	0 (0, 0)	0 (0, 0)	0.511	0 (0, 0)	0 (0, 0)	1
PT < 3.9 (mmol/L)	0 (0, 0)	0 (0, 0)	0.511	0 (0, 0)	0 (0, 0)	0.739
PT in 2.8-10 (mmol/L)	36 (4, 59.5)	72 (47, 89)	0.007	16 (7.25, 57.5)	77.5 (64.25, 89.75)	<0.001
PT in 3.9-10 (mmol/L)	36 (4, 59.5)	71 (41.5, 79)	0.026	16 (7.25, 57.5)	77.5 (64.25, 88.25)	<0.001
PT > 10 (mmol/L)	64 (40.5, 96)	28 (8, 53)	0.007	84 (42.5, 92.75)	22.5 (10.25, 35.75)	<0.001
PT > 13.9 (mmol/L)	27 (2.5, 63)	5 (0, 10.5)	0.019	16.5 (0.75, 37.5)	0 (0, 8.75)	0.035
TNF-*α* (pg/mL)	21.51 ± 14.20	5.00 ± 2.05	0.001	20.42 ± 11.11	5.63 ± 1.55	0.001
IL-6 (pg/mL)	1.42 ± 0.84	0.31 ± 0.23	<0.001	2.19 ± 1.47	0.75 ± 0.65	0.003
8-*iso*-PGF2*α* (pg/mL)	19.90 ± 15.90	11.29 ± 9.03	0.008	23.60 ± 9.90	12.59 ± 6.23	0.002

Abbreviations: FBG: fasting blood glucose; HbA1c: glycosylated hemoglobin; MBG: Mean Blood Glucose; SDBG: Standard Deviation of Blood Glucose; MAGE: Mean Amplitude of Glycemic Excursion; MODD: Absolute Means of Daily Difference; PT: percentage time; TNF-*α*: tumor necrosis factor-*α*; IL-6: interleukin-6; 8-*iso*-PGF2*α*: 8-*iso*-prostaglandin F2*α*. Data shown as mean ± SD or median (first quartile and third quartile).

**Table 3:** Comparison of blood fluctuation, oxidative stress, and inflammatory profile between the dulaglutide and glimepiride groups.

	Baseline		26 weeks after treatment		Δ	
	Dulaglutide	Glimepiride	Dulaglutide	Glimepiride	Dulaglutide	Glimepiride
FBG (mmol/L)	11.12 ± 2.63	9.59 ± 1.32	7.77 ± 1.33	7.45 ± 0.95	−3.35 ± 2.05	−2.14 ± 1.81
HbA1c (%)	8.38 ± 0.93	7.91 ± 0.98	6.68 ± 0.73	6.67 ± 0.74	−1.71 + 0.97	−1.24 ± 1.15
Body weight (kg)	66.85 ± 9.04	66.60 ± 8.56	66.62 ± 8.84	67.80 ± 8.67	0.5 (-1.75, 1.25)	1.75 (-1.75, 4.12)
MBG (mmol/L)	12.35 ± 3.23	11.53 ± 1.88	8.84 ± 2.21	8.59 ± 1.75	−3.51 ± 3.06	−2.94 ± 2.79
SDBG (mmol/L)	2.57 ± 0.74	2.24 ± 0.54	1.98 ± 0.74	2.2 ± 0.92	−0.59 ± 0.84	−0.04 ± 0.68
MAGE (mmol/L)	6.49 ± 2.33	5.35 ± 1.74	5.32 ± 2.6	5.73 ± 2.82	−1.17 ± 3.87	0.38 ± 2.85
MODD (mmol/L)	2 ± 0.59	2.53 ± 0.89	1.71 ± 0.80	1.85 ± 0.75	−0.29 ± 1.03	−0.69 ± 1.02
PT < 2.8 (mmol/L)	0 (0, 0)	0 (0, 0)	0 (0, 0)	0 (0, 0)	0 (0, 0)	0 (0, 0)
PT < 3.9 (mmol/L)	0 (0, 0)	0 (0, 0)	0 (0, 0)	0 (0, 0)	0 (0, 0)	0 (0, 0)
PT in 2.8-10 (mmol/L)	36 (4, 59.5)	16 (7.25, 57.5)	72 (47, 89)	77.5 (64.25, 89.75)	35 (25.5, 54)	51 (9.25, 75)
PT in 3.9-10 (mmol/L)	36 (4, 59.5)	16 (7.25, 57.5)	71 (41.5, 79)	77.5 (64.25, 88.25)	33 (1.5, 51.5)	49 (9.25, 75)
PT > 10 (mmol/L)	64 (40.5, 96)	84 (42.5, 92.75)	28 (8, 53)	22.5 (10.25, 35.75)	-35 (-54.5, -25.5)	-51 (-75, -9.25)
PT > 13.9 (mmol/L)	27 (2.5, 63)	16.5 (0.75, 37.5)	5 (0, 10.5)	0 (0, 8.75)	-20 (-52, -2)	-7.5 (-36.5, 0)
TNF-*α* (pg/mL)	21.51 ± 14.20	20.42 ± 11.11	5.00 ± 2.05	5.63 ± 1.55	−16.51 ± 13.15	−14.79 ± 10.31
IL-6 (pg/mL)	1.42 ± 0.84	2.19 ± 1.47	0.31 ± 0.23	0.75 ± 0.65	−1.11 ± 0.74	−1.44 ± 1.13
8-*iso*-PGF2*α* (pg/mL)	19.90 ± 15.90	23.60 ± 9.90	11.29 ± 9.03	12.59 ± 6.23	−8.61 ± 9.76	−11.01 ± 7.76

Abbreviations: FBG: fasting blood glucose; HbA1c: glycosylated hemoglobin; MBG: Mean Blood Glucose; SDBG: Standard Deviation of Blood Glucose; MAGE: Mean Amplitude of Glycemic Excursion; MODD: Absolute Means of Daily Difference; PT: percentage time; TNF-*α*: tumor necrosis factor-*α*; IL-6: interleukin-6; 8-*iso*-PGF2*α*: 8-*iso*-prostaglandin F2*α*. Data shown as mean ± SD or median (first quartile and third quartile).

## Data Availability

The datasets generated and/or analyzed during the current study are not publicly available due Protecting the Rights and Interests of Subjects but are available from the corresponding authors on reasonable request.
